# The cost‐effectiveness of HIV pre‐exposure prophylaxis in men who have sex with men and transgender women at high risk of HIV infection in Brazil

**DOI:** 10.1002/jia2.25096

**Published:** 2018-03-30

**Authors:** Paula M Luz, Benjamin Osher, Beatriz Grinsztejn, Rachel L Maclean, Elena Losina, Madeline E Stern, Claudio J Struchiner, Robert A Parker, Kenneth A Freedberg, Fabio Mesquita, Rochelle P Walensky, Valdilea G Veloso, A David Paltiel

**Affiliations:** ^1^ The Instituto Nacional de Infectologia Evandro Chagas Fundação Oswaldo Cruz Rio de Janeiro Brazil; ^2^ Medical Practice Evaluation Center Massachusetts General Hospital Boston MA USA; ^3^ Division of General Internal Medicine Massachusetts General Hospital Boston MA USA; ^4^ Harvard University Center for AIDS Research Harvard Medical School Boston MA USA; ^5^ Department of Orthopedic Surgery Brigham and Women's Hospital Boston MA USA; ^6^ Department of Biostatistics Boston University School of Public Health Boston MA USA; ^7^ Biostatistics Center Massachusetts General Hospital Boston MA USA; ^8^ Division of Infectious Disease Massachusetts General Hospital Boston MA USA; ^9^ Department of Epidemiology Boston University School of Public Health Boston MA USA; ^10^ Department of Health Policy and Management Harvard School of Public Health Boston MA USA; ^11^ Division of Infectious Disease Brigham and Women's Hospital Boston MA USA; ^12^ Yale School of Public Health New Haven CT USA

**Keywords:** HIV, pre‐exposure prophylaxis, men who have sex with men, Brazil, cost‐effectiveness

## Abstract

**Introduction:**

Men who have sex with men (MSM) and transgender women (TGW) in Brazil experience high rates of HIV infection. We examined the clinical and economic outcomes of implementing a pre‐exposure prophylaxis (PrEP) programme in these populations.

**Methods:**

We used the Cost‐Effectiveness of Preventing AIDS Complications (CEPAC)‐International model of HIV prevention and treatment to evaluate two strategies: the current standard of care (SOC) in Brazil, including universal ART access (*No PrEP* strategy); and the current SOC plus daily tenofovir/emtracitabine PrEP (*PrEP* strategy) until age 50. Mean age (31 years, SD 8.4 years), age‐stratified annual HIV incidence (age ≤ 40 years: 4.3/100 PY; age > 40 years: 1.0/100 PY), PrEP effectiveness (43% HIV incidence reduction) and PrEP drug costs ($23/month) were from Brazil‐based sources. The analysis focused on direct medical costs of HIV care. We measured the comparative value of *PrEP* in 2015 United States dollars (USD) per year of life saved (YLS). Willingness‐to‐pay threshold was based on Brazil's annual *per capita* gross domestic product (GDP; 2015: $8540 USD).

**Results:**

Lifetime HIV infection risk among high‐risk MSM and TGW was 50.5% with *No PrEP* and decreased to 40.1% with *PrEP*. *PrEP* increased per‐person undiscounted (discounted) life expectancy from 36.8 (20.7) years to 41.0 (22.4) years and lifetime discounted HIV‐related medical costs from $4100 to $8420, which led to an incremental cost‐effectiveness ratio (ICER) of $2530/YLS. *PrEP* remained cost‐effective (<1x GDP) under plausible variation in key parameters, including PrEP effectiveness and cost, initial cohort age and HIV testing frequency on/off PrEP.

**Conclusion:**

Daily tenofovir/emtracitabine PrEP among MSM and TGW at high risk of HIV infection in Brazil would increase life expectancy and be highly cost‐effective.

## 1 Introduction

A growing body of evidence supports the use of tenofovir/emtracitabine pre‐exposure prophylaxis (PrEP) to prevent HIV infection, particularly in high‐risk populations. A 2016 meta‐analysis of 18 PrEP‐related studies conducted in high‐risk populations demonstrated that PrEP reduces HIV infection risk in men who have sex with men (MSM), transgender women (TGW), injection drug users, and other high‐risk populations 1. Moreover, pooled results from the same study showed that PrEP did not increase adverse event rates compared to no PrEP, nor did it result in risk compensation among PrEP users. These results support the World Health Organization's (WHO) 2014 recommendation for PrEP use in high‐risk populations 2.

In Brazil, the HIV epidemic is concentrated in vulnerable populations. Incidence in these groups has increased in the past decade, particularly in young adults in the past five years 3. Recent estimates by the Department of STIs, AIDS and Viral Hepatitis of the Brazilian Ministry of Health report an HIV prevalence of 0.4% in the general population 3. This figure is much higher among MSM, ranging from 5.2% to 23.7% 4. In addition to prevalence, HIV incidence is also elevated among MSM, estimated as >6 infections per 100 person‐years in 2006 5. Recent national data suggest that the already severe epidemic among MSM is growing. In 2007, 30.8% of those newly diagnosed with HIV infection reported sex with other men as the mode of HIV acquisition; by 2016, this proportion reached 50.2% 3. HIV risk is even more pronounced among TGW 6; in Rio de Janeiro, Brazil, their HIV prevalence is estimated at 31.2% 7.

Curbing the rising HIV burden among MSM and TGW in Brazil will require the development of targeted prevention programmes, such as an HIV PrEP programme. PrEP efficacy in Brazil was evaluated in an international randomized controlled trial 8, and the potential implementation of PrEP was also assessed in “PrEP Brasil,” a local demonstration project 9. These studies indicated that PrEP use in high‐risk MSM would be clinically beneficial in Brazil, and our goal was to evaluate the long‐term clinical benefits and cost‐effectiveness of such an intervention. Prior modelling studies have evaluated the cost‐effectiveness of PrEP use among MSM and other high‐risk individuals in other countries 10, 11. Ours is a nation‐specific analysis of daily tenofovir/emtracitabine PrEP use in MSM and TGW at high risk of HIV in Brazil using the best available epidemiological, clinical, and economic data.

## 2 Methods

### 2.1 Analytic overview

We used the Cost‐Effectiveness of Preventing AIDS Complications (CEPAC)‐International model, a widely published model of HIV disease and treatment 12, 13, 14, to project the clinical benefits and economic impact of daily tenofovir/emtricitabine (TDF/FTC)‐based PrEP until age 50 for adult HIV‐uninfected (age ≥18 years) MSM and TGW at high risk of HIV infection. We examined two strategies: standard of care with no PrEP (*No PrEP*) and standard of care with PrEP (*PrEP*). Key outcomes included lifetime HIV infection risk, life expectancy (LE), lifetime HIV‐related medical cost, and incremental cost‐effectiveness ratio (ICER) in dollars per year of life saved (YLS). We conducted sensitivity analyses to examine the robustness of our results to parameter variation. We measured the comparative value of *PrEP* in 2015 United States dollars (USD) per YLS. In conformity with accepted practice 15, all economic outcomes were assessed from the perspective of the Brazilian National Health System. We applied widely accepted conventions with regard to discounting, accounting for time preferences by weighing costs and health outcomes less heavily the further into the future they occur. Economic outcomes were discounted at an annual rate of 3%, while clinical outcomes were reported on both a discounted and undiscounted basis. We excluded medical care costs not related to HIV/AIDS. We defined a strategy as “cost‐effective” if its ICER was less than the Brazilian annual *per capita* gross domestic product (GDP; 2015: $8540 USD).

### 2.2 Model overview

#### 2.2.1 Disease module

CEPAC‐I is a state‐transition Monte Carlo simulation model of HIV infection, detection, disease and treatment. All individuals are tracked from model entry until death, regardless of HIV status. The Disease module of CEPAC‐I simulates disease treatment and progression among HIV‐infected individuals. Upon infection, individuals enter the Disease module of CEPAC‐I, receive an initial CD4 count, and experience disease progression according to user‐defined HIV‐related opportunistic infection (OI) risks and mortality. Model users can define a wide variety of screening and treatment parameters, including HIV screening practices (occasional or routine testing), ART efficacy and toxicity, HIV care practices regarding clinic visits and laboratory monitoring, as well as OI prophylaxis and treatment. Successful ART decreases HIV viral load and increases CD4 count, which leads to reduced mortality from OIs or chronic AIDS. The model also tracks costs for HIV treatment, medication and monitoring; non‐HIV‐related costs are excluded.

#### 2.2.2 HIV testing and PrEP module

In this module, uninfected individuals are assigned initial characteristics, including age and sex at birth (male only for this analysis), and then face user‐specifiable monthly probabilities of HIV infection that vary by age. PrEP is simulated as a reduction in HIV infection probability and has associated medication (TDF/FTC) and HIV testing costs. Individuals can be tested for HIV in two ways: through “background” HIV testing randomly occurring at a specific monthly probability, or through more frequent, regular testing. PrEP is discontinued either when an individual reaches age 50 or if an individual is infected with HIV and detected through testing or a clinically observed OI; upon detection, individuals transition to the Disease Module described above. Key parameters pertaining to the implementation of PrEP are provided in Table 1 and in the Supplementary Materials. A comprehensive technical description of the CEPAC Model is available online at http://www.massgeneral.org/mpec/cepac/. Specifically, the PrEP model description can be found at http://www.massgeneral.org/MPEC/Assets/Files/UpdatedFiles/Pre-Exposure%20Prophylaxis%20_PrEP_.pdf.

**Table 1:** Select model input parameters

Variable	Base case value	Range in sensitivity analysis	References
Baseline cohort characteristic			
Age, years, mean (SD)	31.4 (8.4)	26.4 to 36.4	40
Annual HIV incidence, by age, infections/100 PY			
≤40 years	4.3	2.1 to 6.4	21
>40 years	1	0.1 to 2.0	21
PrEP characteristics			
PrEP effectiveness, % incidence reduction	43.2	21.6 to 64.8	
PrEP efficacy	96		23, 24
PrEP uptake	61		17
PrEP adherence	73.9		22
HIV testing characteristics			
Frequency of HIV test receipt on PrEP, tests/y	3	2 to 4	41
Background testing rate, tests/100 PY	4.4	2.2 to 11.4	42
Clinical characteristics post‐HIV infection			
Acute CD4 count, cells/µL, mean (SD)	559 (236)	419 to 699	18, 19
ART characteristics			
Initial first‐line suppression, %	90	85 to 95	43
Rate of virologic failure, instances/100 PM	0.2		44, 45
Increase in CD4 count after 48 weeks on suppressive ART, cells/µL, mean (SD)	196 (49)		43
PrEP‐associated costs			
PrEP drug cost, $/year	270^a^	135 to 405*	25
HIV test cost, $/test	1.57^b^	0.79 to 3.14	46
Clinic visit cost, $/visit	3.73		47
Creatinine testing cost, $/year	0.69		47
Antiretroviral therapy cost, $/year			
First line: EFV+TDF+3TC	120^a^	72 to 120	46
Second line: LPV/r+TDF+3TC	932	559 to 932	46
Third line: RAL+DRV/r+2 NRTI	6119	3671 to 6119	46
Fourth line: ETR+PI/r+2 NRTI	5549	3330 to 5549	46
Fifth line: MVC+PI/r+2 NRTI	3558	2135 to 3558	46
HIV viral load test, per test, $	14.36	7.18 to 21.54	48
CD4 count test, $/test	13.57	6.79 to 20.36	48
Routine care cost conditional on CD4 count, $/month	3.88 to 43.66	0.5x to 1.5x base case costs	47
Annual discount rate, %	3		15

SD, standard deviation; MSM, men who have sex with men; TGW, transgender women; HIV, human immunodeficiency virus; PY, person‐years; PrEP, pre‐exposure prophylaxis; ART, antiretroviral treatment; PM, person‐months; EFV, efavirenz; TDF, tenofovir disoproxil fumarate; 3TC, lamivudine; LPV/r, lopinavir/ritonavir; RAL, raltegravir; DRV/r, darunavir/ritonavir; NRTI, nucleoside reverse transcriptase inhibitor; ETR, etravirine; PI/r, protease inhibitor/ritonavir; MVC, maraviroc.

a In the Supplementary Material, we consider a range of PrEP drug costs from 0.5x to 1.5x and 2x the base case value.

b First‐line ART drug costs assume fixed‐dose, generic combinations of TDF + EFV + 3TC. PrEP drug costs, in contrast, assume branded formulations of TDF/FTC.

Applies only to HIV testing in the *PrEP* strategy since background testing rate/cost are identical in both the *No PrEP* and *PrEP* scenarios.

### 2.3 Cohort and strategies

We simulated HIV‐uninfected MSM and TGW at high risk of HIV infection in Brazil, modelling them as one population. While TGW experience a higher disease burden than MSM in Brazil 7, we combined the two populations because a PrEP programme in Brazil would likely target both. In the *No PrEP* strategy, individuals faced a monthly risk of HIV infection and were randomly tested for HIV at a rate of 4.4% per year (corresponding to a ~50% expected probability of being tested at least once over a lifetime) as per testing rates observed in this population in Brazil. We examined higher background testing rates in sensitivity analyses. In the *PrEP* strategy, HIV‐uninfected individuals received PrEP from simulation start, HIV testing every four months, and annual creatinine testing until HIV infection and subsequent detection if infected. Upon diagnosis, all HIV‐infected and detected individuals in both strategies received HIV treatment concordant with Brazil's guidelines, including immediate ART initiation regardless of CD4 count 16.

### 2.4 Model input data

#### 2.4.1 Demographic characteristics and natural history

Demographic data used to characterize the simulated cohort were based on PrEP Brasil, a multicentre, open‐label PrEP demonstration project assessing PrEP implementation for MSM and TGW at high risk of HIV infection within the Brazilian public health system 9, 17. For the purposes of the present analysis, we followed the definitions adopted in the PrEP Brasil study and defined high risk as any of the following behaviours in the prior 12 months: condomless anal sex with ≥2 partners, ≥2 episodes of anal sex with an HIV‐infected partner, or history of STD diagnosis. Individuals entering the simulation were assigned an age based on a random draw from a distribution with mean 31.4 years and standard deviation 8.4 years. Individuals became eligible for PrEP when they attained 18 years of age. Mean CD4 count at HIV infection was 559/μL 18, 19. Monthly OI risks, as well as mortality risks attributable to HIV, were derived from the HIV‐infected cohort of the Instituto Nacional de Infectologia Evandro Chagas, Fundação Oswaldo Cruz, in Rio de Janeiro, Brazil 20.

#### 2.4.2 HIV incidence rate

HIV incidence rates without PrEP were 4.3 per 100 PY (<40 years) and 1.0 per 100 PY (≥40 years) 21.

#### 2.4.3 PrEP effectiveness

We defined PrEP effectiveness as a multiplicative composite of three component factors:

- PrEP uptake (i.e. the proportion of the eligible population who accepted the offer of PrEP). We used uptake result from PrEP Brasil of 60.9% 17.
- Adherence (i.e. the proportion of those receiving PrEP whose pharmacokinetic profiles are consistent with 4 or more doses/week). We used week‐48 adherence estimate from PrEP Brasil of 73.9% 22.
- Efficacy (i.e. the performance of PrEP in idealized settings where uptake and adherence can be assumed to be close to 100%). We used efficacy estimates of 96% as derived by a pharmacokinetic model and recently confirmed with directly observed therapy 23, 24.

We multiplied these point estimates (60.9%*73.9%*96.0%) to get a base case effectiveness value of 43.2%. We then conducted sensitivity analyses on this base case estimate to explore the effects of uncertainty in the uptake of PrEP, the adherence to PrEP, and the pharmacologic efficacy of FTC/TDF. We considered PrEP effectiveness values ranging from 0.5 to 1.5 times the base case (21.6% to 64.8%) in order to ensure a wide interval for the effectiveness parameter.

#### 2.4.4 ART regimens and laboratory monitoring

As per Brazilian national guidelines 16, five sequential ART lines were available in the simulation: first‐line ART included efavirenz, tenofovir and lamivudine, second‐line was protease inhibitor‐based, and the remaining three salvage lines included integrase inhibitors and second‐generation non‐nucleoside reverse transcriptase inhibitors. A recent meta‐analysis showed that most cases of drug‐resistant HIV infection occurred among PrEP users who initiated PrEP while acutely HIV infected, and that the incidence of acquiring drug‐resistant HIV during PrEP use was negligible 1; we therefore assumed PrEP users who became HIV infected used the same ART lines as those who did not use PrEP. Moreover, we did not consider PrEP‐induced ART resistance in the base case *PrEP* scenario though its impact was explored in sensitivity analyses. HIV‐infected people were monitored with CD4 tests every six months and HIV RNA two months after initiation of each ART line and every six months thereafter.

#### 2.4.5 Costs

The largest component of PrEP costs was the cost of tenofovir/emtricitabine, which we assumed to be $22.5/month in the base case, as per official contract by the Brazilian Ministry of Health 25. In the Supplementary Material, we also report on 0.5x ($11.25/month) given that lower costs have been reported in the PAHO strategic fund website, as well as upper values of 1.5x ($33.75/month) and 2x ($45/month). We also factored in PrEP programme costs, including clinic visits/counselling every four months ($3.73), HIV testing every four months ($1.57), and annual creatinine testing ($0.69). Since the Brazilian NHS makes no provision for an incremental adherence counselling intervention alongside the PrEP programme, we assume that the costs of adherence counselling are included in the costs of the clinic visit every four months.

HIV tests, administered every four months to persons receiving PrEP, cost $1.57 per test. CD4 and HIV viral load test costs ($13.57 and $14.36) were from the Department of STIs, AIDS and Viral Hepatitis, Ministry of Health (Table 1). Annual ART costs ranged from $120 for first‐line ART to $6119 for third‐line ART; a genotype resistance testing cost of $132 was applied every time an ART regimen was changed. OI treatment costs were from Instituto Nacional de Infectologia Evandro Chagas, Fundação Oswaldo Cruz.

### 2.5 Sensitivity analyses

To assess the robustness of our results, we examined the impact of varying key inputs over a wide range of values; these included average cohort age at model entry, PrEP effectiveness and cost, HIV incidence, age at PrEP discontinuation (including the possibility of lifetime PrEP), and HIV background testing rate. We also examined the impact of ART resistance in a sensitivity analysis where we reduced the efficacy of first‐ and second‐line ART by 10% for patients who acquired HIV while on PrEP. Although some studies have suggested an increase in adverse events (e.g. renal toxicity 26 and reduction in bone mineral density 27) for individuals using tenofovir‐based PrEP, a recent meta‐analysis showed that adverse event rates were not different for those on and off PrEP in 10 placebo‐controlled randomized trials 1. For this reason, we did not include the risk of PrEP‐related adverse events in the base case scenario. Finally, we considered multi‐way analyses to examine outcomes influenced by simultaneous variation in those variables found to be most influential in the one‐way sensitivity analyses described previously.

## 3 Results

### 3.1 Base case

#### 3.1.1 Clinical

Compared to *No PrEP*,* PrEP* increased remaining undiscounted (discounted) life expectancy from 36.8 (20.7) years to 41.0 (22.4) years, or by 4.2 (1.7) years (Table 2).

**Table 2:** Base case results of analysis of PrEP cost‐effectiveness in Brazil

	No PrEP	PrEP
Undiscounted per‐person life expectancy^a^, y	36.8	41.0
Five‐year HIV infection risk^b^, %	16.2	9.7
Lifetime HIV infection risk^b^, %	50.5	40.1
Five‐year averted HIV infections^c^, %	–	6.5
Lifetime averted HIV infections^c^, %	–	10.5
Five‐year HIV‐attributable deaths^d^, %	1.3	0.3
Lifetime HIV‐attributable deaths^d^, %	22.3	11.1
Undiscounted five‐year cost^e^, $	50	940
Discounted five‐year cost^e^, $ (3%)	50	890
Undiscounted lifetime cost^e^, $	10,910	19,070
Discounted per‐person life expectancy^a^, y (3%)	20.7	22.4
Discounted lifetime cost^e^, $ (3%)	4100	8420
ICER, Δcost/ΔLE	–	2530

PrEP, pre‐exposure prophylaxis; y, years; HIV, human immunodeficiency virus; ICER, incremental cost‐effectiveness ratio measured by change in 2015 US dollars (Δcost) per change in life expectancy (ΔLE).

a Life expectancy was defined from start of simulation.

b HIV infection risk was calculated by dividing the number of infections by the initial cohort size.

c Averted HIV infections were calculated by subtracting the number of infections in the *No PrEP* strategy by the number of infections in the *PrEP* strategy.

d HIV attributable death was based on the number of deaths due to HIV related causes among those who were HIV‐infected.

e Cost outcomes are rounded to the nearest $10.

*PrEP* reduced per‐person lifetime (and five‐year) HIV infection risk from 50.5% (16.2%) to 40.1% (9.7%). *PrEP* also increased the speed with which new cases of HIV infection were detected and linked to care. Average CD4 count at detection was 432 cells/mL under *PrEP* and 238 cells/mL under *No Prep*. Owing to both its prevention effects and its rapid linkage of breakthrough infections to care, *PrEP* reduced the probability of dying of HIV‐related causes from simulation start from 1.3% to 0.3% over five years, and from 22.3% to 11.1% over a lifetime (Table 2).

#### 3.1.2 Costs

**Figure 1:**
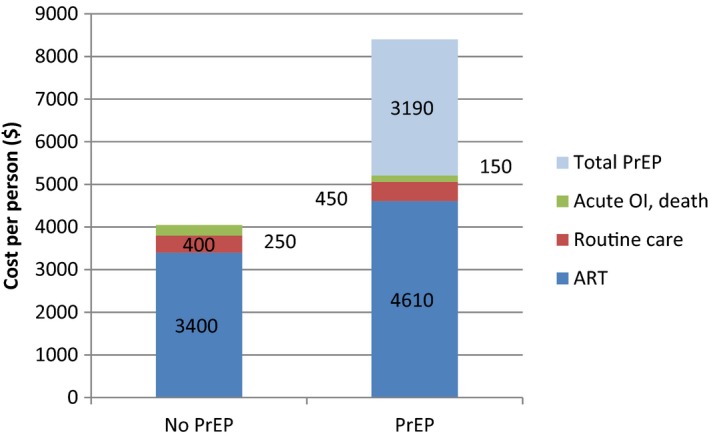
Strategy‐specific cost breakdown. Average discounted per‐person cost of *No PrEP* (left) and *PrEP* (right) strategies stratified by component: ART for treatment; routine care; opportunistic infections and death; and PrEP (drug and monitoring). PrEP: pre‐exposure prophylaxis, OI: opportunistic infection, ART: antiretroviral therapy. Note that background testing costs remain the same under both strategies and are not reported here.

Total costs over five years under *PrEP* are $890/person compared to $47/person for *No PrEP*. Over a lifetime, costs under the *PrEP* strategy are more than double those of *No PrEP* ($8420 vs. $4100 per person). Thirty‐eight percent of the costs under *PrEP* are attributable to prophylaxis medication and testing (Figure 1). Again, as shown previously, although it reduces lifetime risks of HIV infection, *PrEP* also produces more rapid detection and linkage to care and, consequently, expected lifetime costs of HIV treatment (ART and routine care) are higher in the *PrEP* arm than in the *No PrEP* arm. Overall outlays for ART, for example, increase from $3400 to $4610 per person. Because new infections are detected earlier, the costs of OI treatment are lower with *PrEP*.

#### 3.1.3 Cost‐effectiveness

Discounted lifetime per‐person HIV‐related medical costs increased from $4100 with *No PrEP* to $8420 with *PrEP* (Table 2). The ICER of *PrEP* compared to *No PrEP* was $2530/YLS, ~30% of Brazil's 2015 GDP *per capita* of $8540.

### 3.2 Sensitivity analyses

**Figure 2:**
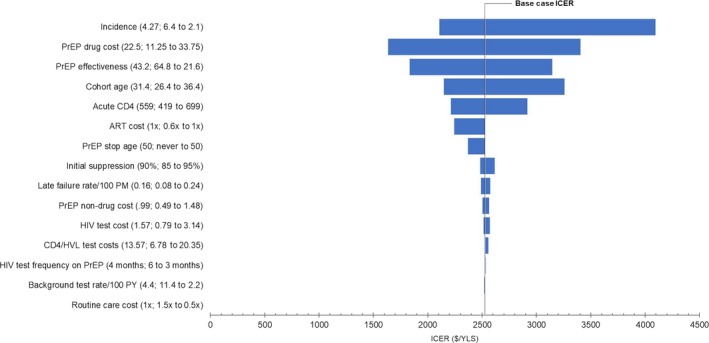
One‐way sensitivity analyses. The tornado diagram displays the sensitivity of the incremental cost‐effectiveness ratio (ICER) of *PrEP* compared to *No PrEP* to variation in a large number of input parameter values. For each input parameter on the vertical axis, base case values are listed first, followed by the range considered in sensitivity analysis. The range values are ordered: the value yielding the lowest ICER in sensitivity analysis is listed first; the value yielding the highest ICER in sensitivity analysis is listed second. GDP: gross domestic product *per capita*, PrEP: pre‐exposure prophylaxis, ART: antiretroviral therapy.

We re‐evaluated our assessment using the data ranges specified in Table 1. Our overall finding was that *PrEP* remains cost‐effective in the face of all plausible uncertainty in the input parameters (Figure 2). Importantly, variation in the ART‐related parameters such as annual ART cost per HIV‐infected person, initial suppression of ART regimens, and CD4 and viral load monitoring costs, as well as others, had minimal impact on the ICER for *PrEP*. Similarly, the cost and frequency of HIV testing – whether as a component of the *PrEP* intervention or as background screening in the at‐risk population – had only minimal impact on the ICER for *PrEP* and no impact on the overall cost‐effectiveness finding. The ICER was virtually unchanged (varying <$200/YLS) when we modified the baseline PrEP duration assumption from discontinuation at age 50 to lifetime use. When we varied average *PrEP* start age between 26.4 and 36.4, the variation in the ICER for *PrEP* amounted to ~$1000/YLS. When we assumed that PrEP‐induced resistance would cause first‐ and second‐line ART to be 10% less effective, the ICER of *PrEP* compared to *No PrEP* increased by $500/YLS, to $3100/YLS. There was no substantial change in the ICER for *PrEP* when we lowered the minimum cohort age from 18 to 15. In the subsections that follow, we note a small number of instances where varying the input assumptions had an informative qualitative effect on the output.

#### 3.2.1 PrEP drug cost

At higher PrEP drug costs, such as $33.75/month (1.5x base case cost), *PrEP* was still cost‐effective: $3300/YLS. *PrEP* remained cost‐effective until PrEP drug costs exceeded $100/month.

#### 3.2.2 HIV incidence

Varying the HIV incidence in the population over its plausible range, from 2.1 infections per 100 PY to 6.4 infections per 100 PY, resulted in ICERs of $2100/YLS to $4100/YLS. *PrEP* was no longer cost‐effective at incidence rates as low as 0.9 per 100 PY (0.2x base case incidence). At very high incidence of 21 infections per 100 PY (5x base case incidence), *PrEP*'s ICER was lower, at $2070/YLS.

#### 3.2.3 PrEP effectiveness

Assuming effectiveness at 0.5 (21.6%) and 1.5 (64.8%) times the base case value (43.2%) led to ICERs of $3140/YLS and $1830/YLS respectively. PrEP‐associated costs accounted for a larger proportion of total strategy cost as effectiveness increased, both because other costs (such as routine care costs and ART costs) decreased and because duration of PrEP use increased.

#### 3.2.4 PrEP effectiveness, HIV incidence and PrEP drug cost

**Figure 3:**
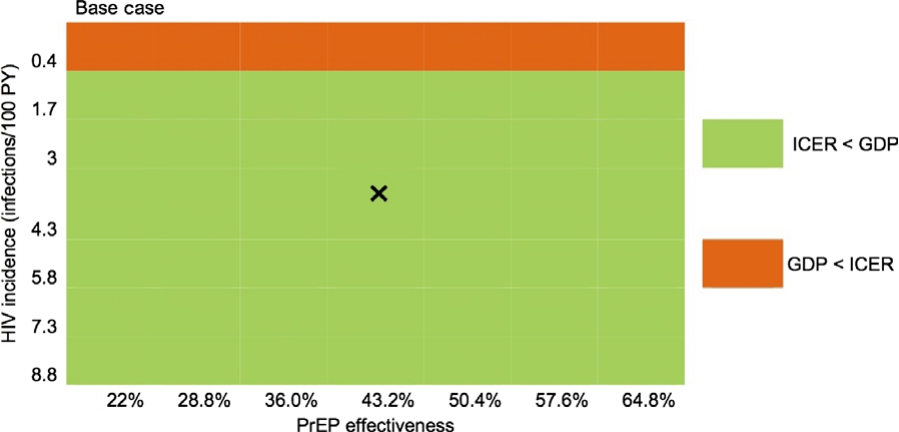
Sensitivity analysis: PrEP effectiveness and HIV incidence. A heat map displays the incremental cost‐effectiveness ratio (ICER) of *PrEP* compared to *No PrEP* as PrEP effectiveness and HIV incidence are simultaneously varied. PrEP effectiveness increases from left to right across the horizontal axis; HIV incidence decreases up the vertical axis. Combinations of PrEP effectiveness and HIV incidence that result in *PrEP* being cost‐saving, cost‐effective, and not cost‐effective compared to *No PrEP* are displayed in blue, green, and orange, respectively. The boldface X in the figure denotes the base case values for PrEP effectiveness and HIV incidence. PrEP: pre‐exposure prophylaxis, GDP: gross domestic product *per capita*.

We simultaneously varied PrEP effectiveness, HIV incidence and PrEP drug cost across wide ranges (Figure 3 and Figure S1). *PrEP* remained cost‐effective compared to *No PrEP* in all scenarios with incidence ranging from 1.7 to 8.8 infections per 100 PY, effectiveness ranging from 22% to 64.8%, and drug cost of $22.5 or $33.75/month. At base case cost ($22.5) *PrEP* was not cost‐effective compared to *No PrEP* when incidence fell to 0.4 infections per 100 PY for effectiveness ranging from 22% to 64.8%.

## 4 Discussion

We evaluated the clinical benefits, costs and cost‐effectiveness of PrEP use to prevent HIV infection in MSM and TGW at high risk of HIV infection in Brazil. We found that a *PrEP* strategy would substantially reduce five‐year and lifetime risk of HIV infection. Compared to standard of care, we found that *PrEP* was highly cost‐effective by GDP standards, with an ICER of $2530/YLS (~30% of Brazil's 2015 *per capita* GDP, $8540). We also found that *PrEP* would remain cost‐effective unless PrEP drug costs exceeded $1200/year ($100/month).

In one‐way sensitivity analyses, we found that varying parameter values within plausible ranges yielded ICERs that were lower than Brazil's GDP *per capita*, suggesting that our conclusions are robust to uncertainty in our input data values. In the base case scenario, *PrEP* was no longer cost‐effective if HIV incidence was less than 0.4 per 100 PY (<10% of base case value). Prior research has suggested that HIV incidence among MSM around the globe far surpasses this threshold, and is likely higher among young MSM 5. For PrEP effectiveness, the lowest value assessed (22% effectiveness) still indicated that *PrEP* was cost‐effective. Even when assuming worst‐case estimates for PrEP drug cost of $45 per month, the ICER of *PrEP* was of $4300 per YLS, half of Brazil's GDP *per capita*. *PrEP* acts both as prevention against new infections and as a detection and linkage tool for the cases of infection that it fails to prevent. Because of this, *PrEP* is creating not only new prevention costs, but also increasing the costs of care. Indeed, our results show that ART costs under *PrEP* are even higher than in the *No PrEP* strategy (Figure 1). Though *PrEP* is more costly than *No PrEP*, both the prevention provided by *PrEP* and the earlier detection and linkage to care for infections that are not prevented leads to more years of life saved and is ultimately cost‐effective.

This is the first analysis to use simulation modelling to estimate the cost‐effectiveness of a PrEP programme in Brazil. One analysis investigating the use of PrEP in Peruvian MSM and TGW found that the highest estimated cost per disability adjusted life‐year averted was below Peru's GDP *per capita*, assuming PrEP use by 20% of MSM and TGW 28. In our analysis, we based PrEP uptake on the results of the PrEP Brasil demonstration project, which showed that 61% of the eligible population chose to initiate PrEP 17. In addition, we assumed adherence to the drugs as observed in this project. We found that with this level of uptake and adherence, PrEP led to a cost per life‐year saved that was well below Brazil's GDP *per capita*. Other analyses examining the value of PrEP among MSM in concentrated epidemics in high‐income countries found that PrEP was cost‐saving or cost‐effective 10, 29, 30. In the United States, providing PrEP to MSM at high risk for HIV infection would yield an ICER of $50,000 per quality adjusted life year, making it cost‐effective by US standards 10. Another analysis considering both the US and Peru looked at main and casual partnerships and found that targeting men in casual partnerships could increase PrEP's efficiency 31. Our results reinforce the value of PrEP for populations at high risk of HIV infection and suggest that one of the challenges to PrEP delivery will be the process of risk assessment by clinicians. In this regard, it is encouraging that an open‐label extension study 32 as well as PrEP Brasil 9 both found that men at higher risk of HIV infection are both more willing to use PrEP and more adherent. Nevertheless, studies measuring behaviour over time show wide variation, such that clinicians will need to continually re‐evaluate high‐risk behaviour 33.

Our results should be interpreted with caution. A finding of cost‐effectiveness indicates a favourable return on investment but cannot definitively address the affordability of *PrEP*. A recent review of PrEP analyses 18 highlights the need for future studies to consider the budget impact of PrEP implementation. Our analysis has a number of other limitations. Though we have used the most recent estimate of HIV incidence derived from a multi‐site international trial, which is consistent with published prevalence estimates 4 and with model derived cumulative risk, HIV incidence estimates for Brazil's MSM and TGW at high risk of HIV population are scarce. We conducted extensive sensitivity analyses and found that our conclusions were robust to HIV incidence; *PrEP* was not cost‐effective when incidence was at 0.4 infections per 100 PY (<10% base case incidence). Nevertheless, *PrEP* implementation and coverage may modify HIV incidence such that research into HIV incidence in different populations will become more important. The *No PrEP* strategy assumed current levels of ART uptake, although it is plausible that Brazil's current test and treat strategy could lead to earlier ART uptake in future years, which in turn could decrease HIV incidence. However, the current HIV care cascade among MSM suggests that MSM and TGW are not experiencing major improvements in access to care in Brazil 5, 34, likely due to high levels of stigma and discrimination. A recent analysis from our cohort suggested that, contrary to what was observed in heterosexual men, the increased risk of AIDS‐related deaths among MSM could not be explained by immunological and clinical factors associated with AIDS mortality, again suggesting that stigma and discrimination might be important barriers to care for this population 35. Indeed, stigma and discrimination could also represent important barriers for PrEP uptake, and, if that were the case, there could be additional costs associated with diminishing barriers and increasing demand that we have excluded. Fortunately, though PrEP‐related stigma has emerged as a social harm that can arise from PrEP research participation/use, the PrEP Brasil study found that only 4.6% of its participants reported social harms 22. In this analysis, we accounted only for first‐generation, age‐dependent HIV transmission to uninfected MSM and TGW at high risk of HIV infection and therefore did not include any second‐order benefits that PrEP will provide. Including secondary transmission would make *PrEP* even more cost‐effective. Our analysis also does not take into consideration the potential savings that could be enjoyed by limiting PrEP use to periods (or ages) when individuals are at highest risk/susceptibility for HIV infection 36, 37. Taking such “seasons of risk” into account could further strengthen the case for expanded PrEP. The present study combined MSM and TGW into one population given the scarcity of data regarding TGW in our setting. These assumptions should be revised and the analysis refined when TGW‐specific data become available, though the high HIV burden among TGW suggests conclusions would not change. We restricted our study population to MSM and TGW aged 18 years or older because this population would likely be the first offered PrEP. However, Brazil's expanding HIV epidemic among MSM younger than 18 years led us to explore a scenario where minimum cohort age was 15 years. While progress has been made to estimate preference‐based weights for health states based on a representative sample of the Brazilian population 38, data are not yet available to support the use of quality‐adjusted life years in this analysis. Readers should note, therefore, that our definition of “cost‐effective” used an ICER measured in dollars per year of life saved and not in more commonly recommended measures that account for the quality of life 39. Finally, the possibility of generic manufacturing of TDF/FTC as well as the adoption of branded drugs for ART (such as the dolutegravir for first‐line regimen 16) could modify the results generated in the present study against and for *PrEP* respectively.

## 5 Conclusion

Our cost‐effectiveness findings support immediate expansion of investment in combination prevention to include PrEP in MSM and TGW populations at the highest risk for HIV infection in Brazil. They also justify further study of the budget impact of PrEP implementation. Our results show that HIV incidence is the major determinant of PrEP cost‐effectiveness and thus merits future monitoring. In the present scenario of increasing HIV incidence among young MSM and TGW, PrEP implementation, in the context of a combination prevention framework, including HIV testing as well as ART adherence interventions, will be cost‐effective.

## Competing interests

The authors declare no competing interests.

## Authors' Contributions

PML, BG, KAF, RPW, VGV and ADP contributed to the conception of the study design. PML, BO, RLM, MES, RAP and ADP conducted the analysis. PML, BG, CJS, FM and VGV acquired data. PML, BO, RLM, EL, MES, RAP, KAF, RPW and ADP analysed and interpreted the data. PML, BO and ADP wrote the first manuscript draft. PML, BO, BG, RLM, EL, MES, CJS, RAP, KAF, FM, RPW, VGV and ADP edited the manuscript.

## Supporting information


**Figure S1.** Three‐way sensitivity analysis: HIV incidence (range), PrEP effectiveness (range) and PrEP drug cost (A: $11.25, B: $33.75 and C: $45).
**Table S1.** Results of analysis of PrEP cost‐effectiveness in Brazil including a 5% discount rate.
**Table S2.** Model Input Parameters for PrEP.
