# Supplementary material for: The cost‐effectiveness of HIV pre‐exposure prophylaxis in men who have sex with men and transgender women at high risk of HIV infection in Brazil
Source: J Int AIDS Soc. 2018 Mar 30;21(3):e25096. doi: 10.1002/jia2.25096 (PMC5878414; doi:10.1002/jia2.25096)
Supplement: Supplementary file 1 — Figure S1. Three‐way sensitivity analysis: HIV incidence (range), PrEP effectiveness (range) and PrEP drug cost (A: $11.25, B: $33.75 and C: $45). Table S1. Results of analysis of PrEP cost‐effectiveness in Brazil including a 5% discount rate. Table S2. Model Input Parameters for PrEP. [file JIA2-21-e25096-s001.docx]

**Supplementary Material**

**The cost-effectiveness of HIV pre-exposure prophylaxis men who have sex with men and transgender women at high risk of HIV infection in Brazil**

**Luz et al.**

**Figure 1. Three-way sensitivity analysis: HIV incidence (range), PrEP effectiveness (range) and PrEP drug cost (A: $11.25, B: $33.75 and C: $45).**

**Table 1. Results of analysis of PrEP cost-effectiveness in Brazil including a 5% discount rate.**

|  | No PrEP | PrEP |
| --- | --- | --- |

| **Undiscounted per-person life expectancy ^a^, y** | 36.8 | 41 |
| --- | --- | --- |
| **5-year HIV infection risk ^b^, %** | 16.2 | 9.7 |
| **Lifetime HIV infection risk ^b^, %** | 50.5 | 40.1 |
| **5-year averted HIV infections ^c^, %** | - | 6.5 |
| **Lifetime averted HIV infections ^c^, %** | - | 10.5 |
| **5-year HIV-attributable deaths ^d^, %** | 1.3 | 0.3 |
| **Lifetime HIV-attributable deaths ^d^, %** | 22.3 | 11.1 |
| **Undiscounted 5-year cost, $** | 50 | 940 |
| **Discounted 5-year cost, $ (3%)** | 50 | 890 |
| **Discounted 5-year cost, $ (5%)** | 44 | 850 |
| **Undiscounted lifetime cost, $** | 10,900 | 19,100 |
|  |  |  |
| **Discounted per-person life expectancy ^a^, y (3%)** | 20.7 | 22.4 |
| **Discounted per-person life expectancy ^a^, y (5%)** | 15.5 | 16.6 |
| **Discounted lifetime cost, $ (3%)** | 4,100 | 8,400 |
| **Discounted lifetime cost, $ (5%)** | 2,400 | 5,600 |
| **ICER, Δcost/ΔLE (3%)** | - | 2,500 |
| **ICER, Δcost/ΔLE (5%)** | - | 3,000 |

PrEP: pre-exposure prophylaxis; y: years; ICER: incremental cost-effectiveness ratio measured by change in 2015 US dollars (Δcost) per change in life expectancy (ΔLE)

^a^ Life expectancy was defined from start of simulation.

**Table 2. Model Input Parameters for PrEP.**

| Variable | **Base Case Value** | **Reference** |
| --- | --- | --- |
| ***No PrEP*** |  |  |
| HIV testing frequency, tests/y | 3 | [27] |
| HIV testing frequency probability | 0 |  |
| HIV testing stop age, mths | none |  |
| ***PrEP*** |  |  |
| HIV testing frequency, tests/y | 3 | [27] |
| HIV testing frequency probability | 1 |  |
| HIV testing stop age, mths | 600 |  |
| **Costs** |  |  |
| PrEP Associated Costs |  |  |
| PrEP drug cost, $/year | 270 | [25] |
| HIV test cost, $/test | 1.57 | [36] |
| Clinic visit cost, $/visit | 3.73 | [37] |
| Creatinine testing cost, $/year | 0.69 | [37] |
| Undetected HIV+ death due to Chronic AIDS or non-AIDS causes cost, $/death | 879.42 | IPEC data and cost calculations |
